# Leveraging celebrity influence for oral cancer prevention and smokeless tobacco cessation: challenges and opportunities in India

**DOI:** 10.3389/fpubh.2025.1531268

**Published:** 2025-07-07

**Authors:** Saravanan Sekaran, Dhanraj Ganapathy, Deepavalli Arumuganainar, Harini Arumugasamy, Vimalraj Selvaraj, Saeed Alassiri, Shahabe Saquib Abullais

**Affiliations:** ^1^Department of Prosthodontics, Saveetha Dental College and Hospitals, Saveetha Institute for Medical and Technical Sciences, Chennai, India; ^2^Department of Periodontics, Saveetha Dental College and Hospitals, Saveetha Institute of Medical and Technical Sciences, Saveetha University, Chennai, India; ^3^Department of Anatomy, Faculty of Allied Health Sciences, Dr. M.G.R Educational and Research Institute, Chennai, India; ^4^Department of Applied Mechanics and Biomedical Engineering, Indian Institute of Technology-Madras, Chennai, India; ^5^Department of Diagnostic Dental Sciences and Oral Biology, King Khalid University, Abha, Saudi Arabia; ^6^Department of Periodontics and Community Dental Science, College of Dentistry, King Khalid University, Abha, Saudi Arabia

**Keywords:** oral cancer, smokeless tobacco, celebrity influence, public health campaigns, surrogate marketing, health advocacy

## Abstract

India bears the highest global burden of oral cancer, with over 83,400 cases reported in 2022. The widespread use of smokeless tobacco and areca nut, particularly in rural and underserved communities, continues to drive this public health crisis. Despite government regulations and awareness campaigns, tobacco consumption remains high due to cultural acceptance, affordability, and accessibility of products like gutkha and khaini. This review examines the role of celebrity endorsements in tobacco cessation efforts, assessing their influence on public perception and behavior. Bollywood actors and sports icons, who hold immense cultural influence in India, have been leveraged in health campaigns to promote tobacco-free lifestyles. While some celebrity-driven initiatives have successfully raised awareness, surrogate marketing remains a critical challenge. Many celebrities indirectly endorse harmful products like pan masala and mouth fresheners, undermining tobacco control efforts. To counter this, stricter policies are needed to regulate celebrity endorsements, ensuring alignment with public health goals. This review highlights key strategies for maximizing the impact of celebrity-led health campaigns while minimizing the risks posed by surrogate marketing. By fostering sustained celebrity engagement and implementing robust policy measures, India can take significant steps toward reducing smokeless tobacco use and preventing oral cancer.

## Introduction

India accounts for the highest number of oral cancer cases linked to the use of smokeless tobacco and areca nut in South Asia. A recent study underscores the ongoing and increasing burden of oral cancer in India, emphasizing the role of lifestyle and socioeconomic factors. There is an urgent need for targeted strategies to reduce risk behaviors, enhance early detection, and tackle disparities to lessen the impact of the disease (1). In 2022, out of the 120,200 global cases, a staggering 83,400 occurred in India. The country’s burden of oral cancer is primarily driven by the widespread consumption of smokeless tobacco products, such as gutkha, khaini, and paan with areca nut, which are culturally ingrained and readily accessible across all socioeconomic groups (2, 3). These products are often chewed, sucked on, or even sniffed, leading to high exposure to carcinogenic substances. The easy availability, affordability, and deeply rooted social acceptance of these habits, particularly in rural and low-income communities, have contributed to India’s disproportionate share of oral cancer cases (4). The high prevalence of tobacco use in both smokeless and smoked forms, coupled with low public awareness of the risks associated with its consumption, has made oral cancer a growing epidemic in the country. The World Health Organization (WHO) has repeatedly emphasized that tobacco use is the single most preventable cause of cancer, and in India, it remains deeply embedded in social and cultural practices. Furthermore, cigarette and bidi smoking continues to thrive, despite numerous anti-smoking campaigns and legislation aimed at reducing tobacco consumption (5). Given the extensive influence of media and popular culture in India, celebrity endorsements and participation in public health campaigns have emerged as effective tools to amplify messages around health and behavioral change. Celebrities, particularly those from the entertainment and sports industries, are revered as cultural icons and hold considerable sway over public opinion (6). Their involvement in health campaigns can not only raise awareness but also play a critical role in reducing the use of tobacco and, consequently, the incidence of oral cancer. This communication explores the role of celebrities in India’s public health campaigns aimed at oral cancer prevention and tobacco cessation, analyzing successful strategies, challenges, and the path forward for more effective outreach.

### Understanding oral cancer in India: a public health crisis

Oral cancer is primarily caused by the prolonged use of tobacco and areca nut, both of which are carcinogenic substances. In India, smokeless tobacco products, often combined with areca nut, are more widely consumed than cigarettes (4, 7). This is particularly evident in rural populations, where tobacco use is often culturally ingrained and less regulated. Moreover, the accessibility and low cost of smokeless tobacco products, along with a lack of adequate health education, have contributed to their widespread use (8). The clinical burden of oral cancer is alarming. Patients often present with advanced stages of the disease due to delayed diagnosis, largely because of low awareness about early symptoms such as persistent mouth ulcers, white or red patches, and difficulty swallowing. The impact on quality of life is severe, as treatments such as surgery, radiation, and chemotherapy can result in significant facial disfigurement, speech difficulties, and compromised nutrition.

**Figure 1:**
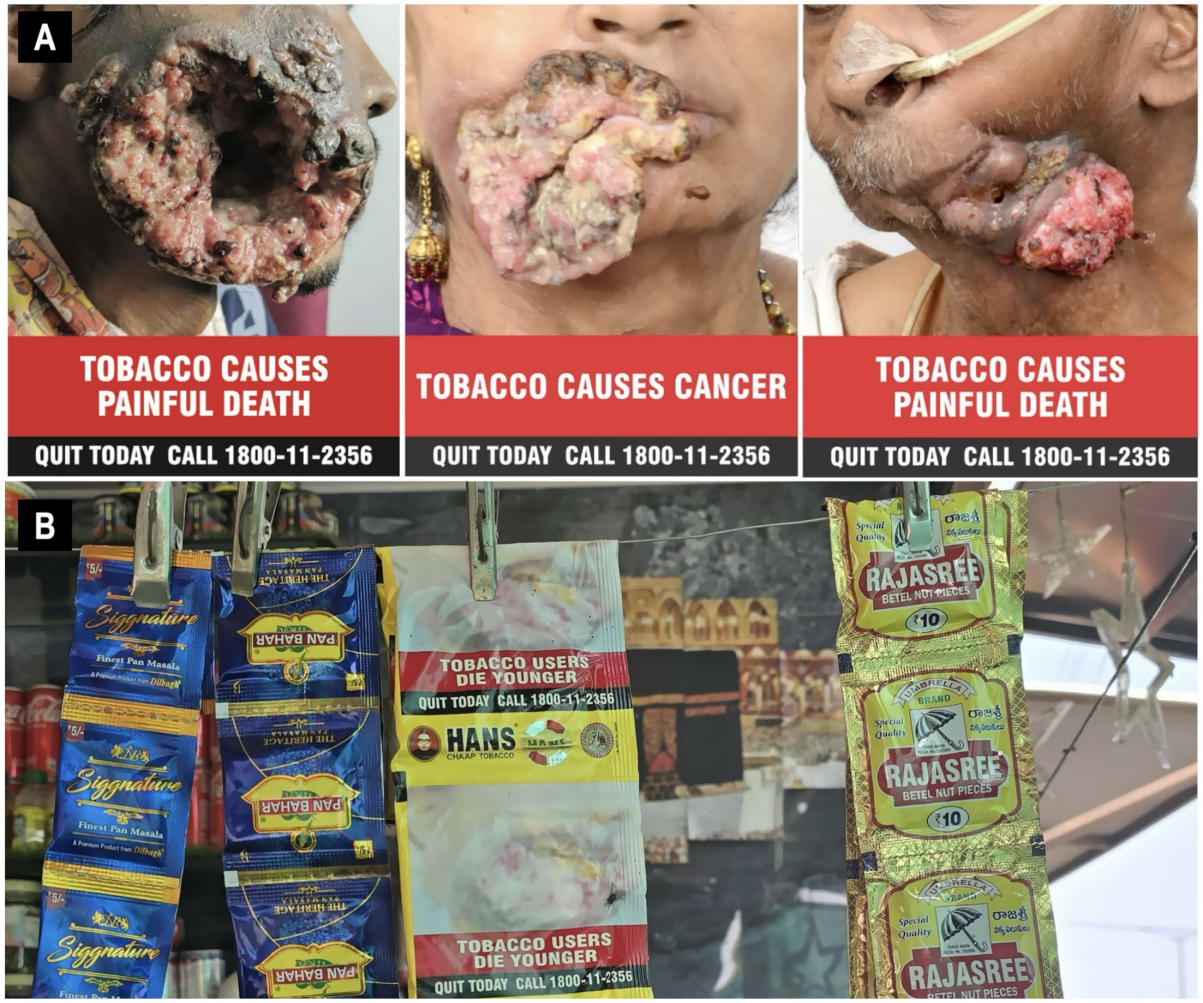
**(A)** Images as per the COTP (Packaging and Labelling) Amendment Rules in India. Clinical photographs of individuals affected by advanced oral cancer resulting from chronic tobacco use. The images are accompanied by public health warnings such as “Tobacco causes painful death” and “Tobacco causes cancer,” along with a national Quitline number (1800-11-2356) to encourage cessation. **(B)** This commercial display of various tobacco products in sachets openly sold in a local shop. Only few brands print mandatory health warnings on the packaging. Health warning can be seen on the Hans packet whereas, signature pan masala does not print these pictures. The easy accessibility and marketing of these products pose significant challenges to tobacco control efforts. Reproduced with permission from “NATIONAL TOBACCO CONTROL PROGRAMME” by the Ministry of Health & Family Welfare Government of India.

The widespread use of smokeless tobacco products, such as gutkha and khaini, combined with poor awareness, delayed diagnosis, and limited access to early screening, has led to an alarming rise in cases. Despite regulatory efforts like the COTPA Act (Table 1) and bans on certain tobacco products, enforcement gaps and cultural normalization of tobacco use continue to fuel this crisis. Addressing oral cancer in India requires a multifaceted approach involving prevention, early detection, robust policy implementation, and community-based education. India’s ban on gutkha, once hailed as a major public health achievement, has been significantly weakened by industry evasion tactics, inadequate enforcement, and pervasive corruption. Manufacturers continue to flourish by exploiting legal loopholes, using strategies like dual packaging and underground distribution networks. As a result, efforts to protect public health, enforce regulations, and curb tobacco use face persistent challenges rooted in deep-seated societal practices. Preventing oral cancer in India, therefore, hinges on reducing tobacco use through public health initiatives, education, and policy. Anti-tobacco legislation such as the Cigarettes and Other Tobacco Products Act (COTPA) of 2003—which includes measures like pictorial health warnings, public smoking bans, and restrictions on advertising—tobacco consumption rates in India remain high, particularly in rural areas where enforcement is inconsistent (9). Public smoking is banned under Section 4 of COTPA, and Section 7 mandates pictorial health warnings covering 85% of tobacco packaging (Figure 1), yet compliance varies widely. These gaps in implementation limit the impact of regulatory measures, necessitating more robust surveillance and community engagement strategies.

**Table 1:** COTPA, 2003 and related rules – summary table.

Section / Rule	Provision / Title	Description / Key points
Section 4	Prohibition of smoking in public places	Smoking is banned in all public places including hospitals, educational institutions, public transport, and workplaces.
Section 5	Prohibition of advertisement of tobacco products	Direct and indirect advertisements of tobacco products are prohibited across all media platforms.
Section 6(a)	Prohibition on sale to minors	Sale of tobacco products to individuals below 18 years of age is prohibited.
Section 6(b)	Prohibition of sale near educational institutions	Tobacco products cannot be sold within a 100-yard radius of any educational institution.
Section 7	Packaging and labeling	Tobacco product packages must carry specified health warnings, including pictorial warnings covering 85% of the package.
Section 8–10	Specification and display of warnings	Specifies the manner, size, and language of health warnings on tobacco packaging.
Section 11	Testing of contents	Mandates testing of nicotine and tar contents by recognized labs and disclosure by manufacturers.
Prohibition Rules	Prohibition on sale of gutkha and pan masala with tobacco (food safety regulations, 2011)	Sale of gutkha and pan masala containing tobacco is banned under FSSAI regulations citing public health safety.
COTPA Amendment Rules, 2015	Strengthening health warnings	Introduced 85% pictorial and textual health warnings on both sides of packaging.
Juvenile Justice Act, 2015	Tobacco sale to minors treated as criminal offense	Selling tobacco to minors is punishable by 7 years imprisonment under this act.
FSSAI Regulation (2011)	Ban on food products containing tobacco	Prohibits the use of tobacco and nicotine as ingredients in any food products, including gutkha.
The Cable Television Network Rules, 1994 (Amended)	Ban on tobacco advertisements in TV, Films, and OTT platforms	Regulates depiction and promotion of tobacco products in visual media through strict content guidelines.

The following table is a concise information based on the information provided in national tobacco control programme website (https://ntcp.mohfw.gov.in/cigarettes_and_other_tobacco_products).

The role of media, particularly celebrity influence, has emerged as a critical avenue for amplifying public health messages and fostering a cultural shift away from tobacco use (10, 11).

### The increasing trend of smokeless tobacco use in India

Globally, approximately 77% of oral cancer cases attributed to smokeless tobacco and areca nut use were among men, with about 92,600 cases, while 23% of cases, or 27,600, were among women. Typically, men have a higher proportion of oral cancer cases caused by these substances than women. However, in regions like southern Africa and Southeast Asia, women surpass men in smokeless tobacco and areca nut consumption, leading to a reversal of this trend, with a higher prevalence of oral cancer among women in these areas (2).

In India, a national cross-sectional household survey portrayed that around 30% of the population either smoked or chewed tobacco (12). Apart from this north eastern states and eastern part of India reports the highest tobacco consumption rates ranging from 35 to 50% (13). The global adult tobacco survey of India concluded that tobacco use is 13.1% higher in rural areas compared to urban areas with khaini being most commonly consumed form (12%) of smokeless tobacco (13–15). In states where smoking was previously more common, stricter smoking bans have led to an increase in the use of smokeless tobacco (16). Higher socioeconomic status is linked to increase use of cigarette while smokeless tobacco consumption (22%) surpassed cigarette smoking (4%) and bidi (17%) (16). In India, a cross-sectional study in Bihar’s villages concluded the highest smokeless tobacco use with 33% and khaini being the dominant choice (57%) (17, 18). Almost 50% of tobacco use begins in childhood or adolescence with increasing prevalence among school students (18%) is alarming (19, 20). Even healthcare professionals are not exempt, with 15% of physicians reported to use tobacco (21, 22). Smokeless tobacco increases the risk of oral cancer by 1.8 to 5.8 times, and esophageal cancer by 2.1 to 3.2 times (23).

**Figure 2:**
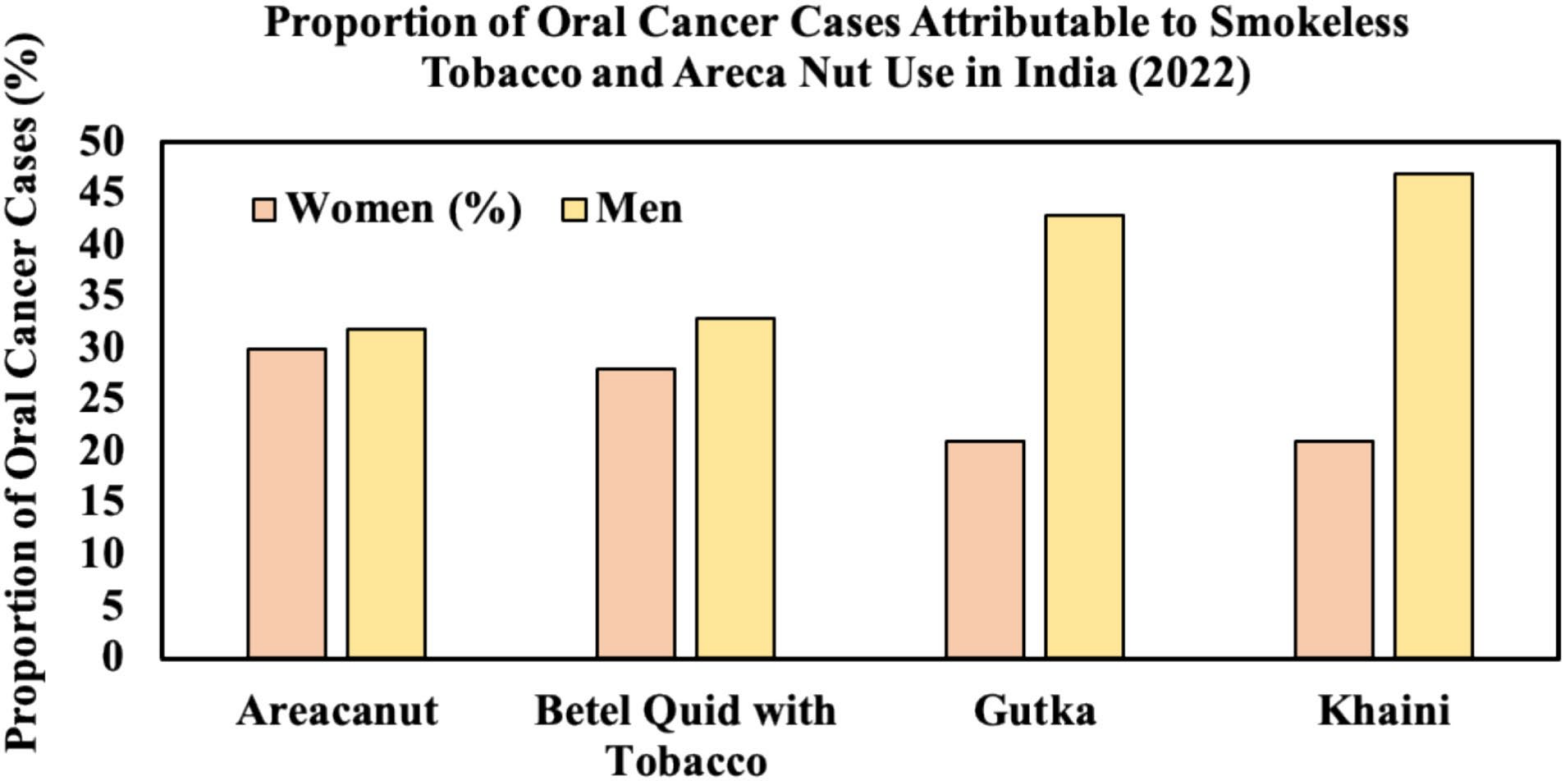
Proportion of oral cancer cases attributable to smokeless tobacco.

The use of areca nut (30%) and betel quid with tobacco (28%) were the most significant contributors to oral cancer cases among women, followed by the consumption of gutka and khaini, each accounting for 21% of cases. Among men, khaini was responsible for the largest proportion of oral cancer cases, at 47%, with gutka being the second highest contributor at 43%. Betel quid mixed with tobacco (33%) and areca nut (32%) were also major causes of oral cancer in men (Figure 2). These statistics underscore the profound impact that smokeless tobacco products and areca nut have on oral cancer rates in India, particularly given their deep cultural roots and widespread availability (2). Areca nut, commonly chewed on its own or combined with other substances like betel leaf and tobacco, is considered a carcinogen by the World Health Organization, yet remains a socially accepted practice in many parts of the country. Women in India often use these products as part of traditional customs, particularly in rural areas, which contributes to the high rates of oral cancer in females (2, 5). Public health campaigns, stronger regulations, and greater awareness are critical in addressing this issue, as both men and women continue to face a growing threat from products that are easily accessible and ingrained in everyday practices.

While smoking-related awareness and advertisements are prevalent in India, there has been significantly less focus on banning smokeless tobacco products. Despite the known health risks of products like khaini, gutka, and betel quid, enforcement and public health campaigns targeting smokeless tobacco remain minimal.

### The influence of celebrities in India: a cultural perspective

Celebrities, particularly Bollywood actors and sports icons, are deeply revered figures in Indian society. Their endorsements hold significant influence over public perception and behavior. Positive involvement of celebrities in anti-tobacco and oral cancer prevention campaigns has successfully increased awareness, especially in rural and underserved populations. High-profile figures like Amitabh Bachchan, Sachin Tendulkar, and Rajinikanth have publicly supported anti-tobacco campaigns, setting an example for their followers and promoting behavior change. Their endorsement of products, social causes, and even political movements can significantly impact public behavior and attitudes. Celebrity-driven campaigns have been linked to increased awareness, engagement, and participation in health-related initiatives. In India, where smokeless tobacco use is deeply ingrained in culture, celebrities have the power to shift social norms by discouraging its use.

Successful campaigns have demonstrated a measurable impact on reducing tobacco consumption, particularly among youth and first-time users. The deep connection between fans and celebrities in India is rooted in a tradition of hero-worship, where celebrities are seen as role models and influencers who transcend the boundaries of entertainment (24).

The advent of mass media, television, and more recently, social media, has further amplified the reach of celebrities. Their ability to connect with audiences across geographic and socioeconomic lines makes them ideal advocates for health campaigns that seek to reach a diverse population. In particular, celebrities can play a crucial role in challenging the social norms and cultural practices that promote tobacco use, particularly in rural and underserved areas. By leveraging their influence, celebrities can increase the visibility of public health messages, encourage healthier behaviors, and reduce the stigma associated with seeking help for tobacco addiction. Their endorsement lends credibility to health campaigns, particularly when they share personal experiences or actively participate in the initiatives. Moreover, the endorsement of celebrities can also generate media coverage and public discourse, ensuring that the health message remains a topic of conversation (10).

### Surrogate marketing of tobacco products

**Figure 3:**
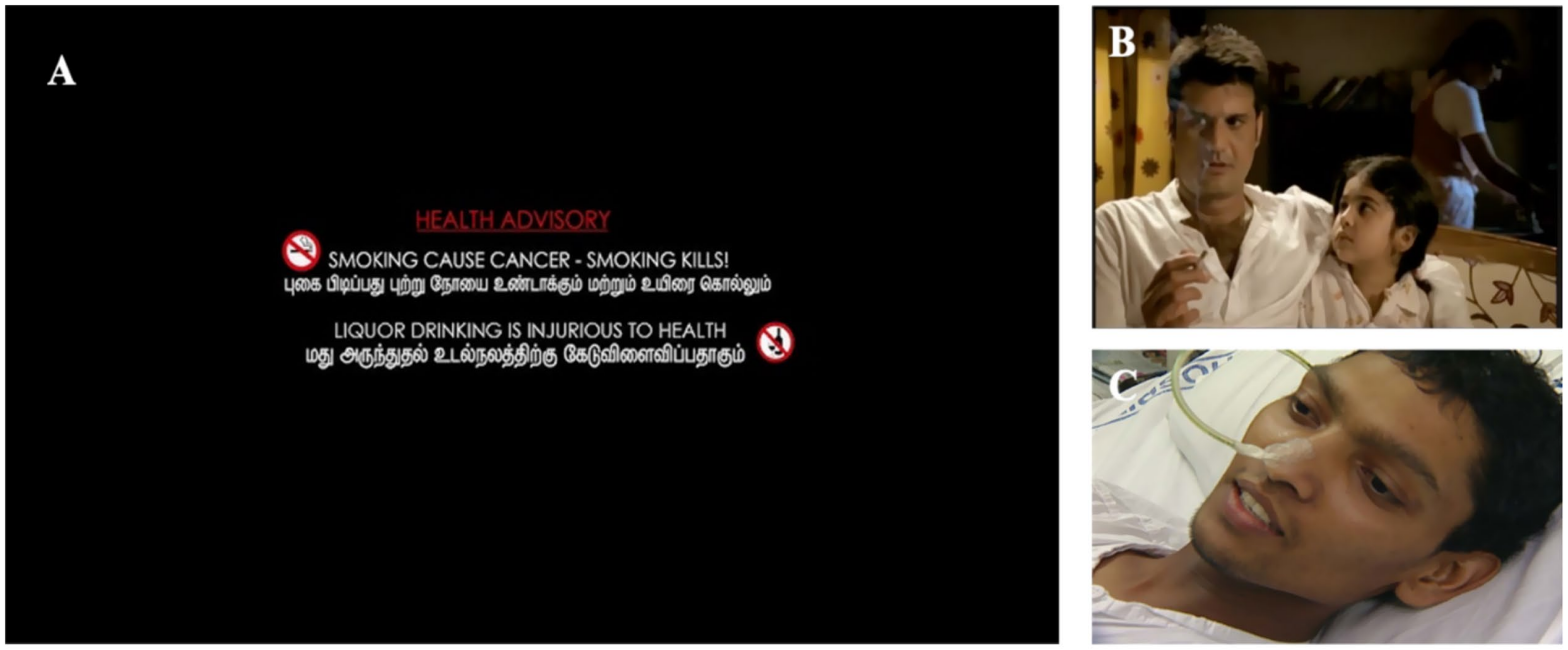
**(A)** The health advisory notification image. Reproduced from postermywall.com. **(B,C)** Screenshot from the anti-tobacco spot titled “Child” under the COTPA rules and ‘Mukesh’ Television announcement, respectively. It is mandatory for the spot to be screened in movies whenever a smoking scene is displayed. This emphasizes the health hazards of smoking. This Public Service Announcement (PSA) has been developed by the Ministry of Health and Family Welfare, India. **(A)** XXX Screenshots from: **(B)** “Ministry of Health and Family Welfare: Anti Tobacco spot “child” by PIB India **(C)** “India - Mukesh: Smokeless Tobacco Campaign (Hindi) - Testimonial” by Vital Strategies.

Despite the Indian government’s numerous health advisories, cigarettes and pan based products are still readily available at convenience stores. Most people recognize anti-smoking ads like the one featuring Mukesh or the ad where a daughter encourages her father to quit smoking (Figures 3A-C). However, Bollywood films and stars often undermine these efforts. The World Health Organization estimates that over three-quarters of Bollywood films depict tobacco use, with cigarettes featured in 72% of scenes (25). A 2012 study also revealed a connection between watching Bollywood portrayals of tobacco use and increased smoking among Indian adolescents (26). Moreover, tobacco companies exploit surrogate advertising to promote their products on television.

Surrogate marketing is a tactic used by companies to promote restricted products, such as tobacco, under the guise of other legal products. In India, despite strict regulations on advertising tobacco, brands often advertise products like mouth fresheners, pan masala, or other similar items, which are visually or textually linked to tobacco products (27). These advertisements typically feature celebrities, including popular Bollywood actors, to appeal to a wide audience. The real intent of such marketing is to indirectly promote tobacco consumption, often targeting vulnerable groups such as youth. The Cigarettes and Other Tobacco Products Act (COTPA), enacted in 2003, prohibits direct advertising of tobacco products in India (28). However, companies circumvent this by advertising “look-alike” products with the same branding, logos, or packaging used for their tobacco items. This creates brand recall and familiarizes consumers with the tobacco product under the disguise of an innocuous item. For instance, brands use pan masala or flavored mouth fresheners as a surrogate to indirectly promote chewing tobacco.

**Figure 4:**
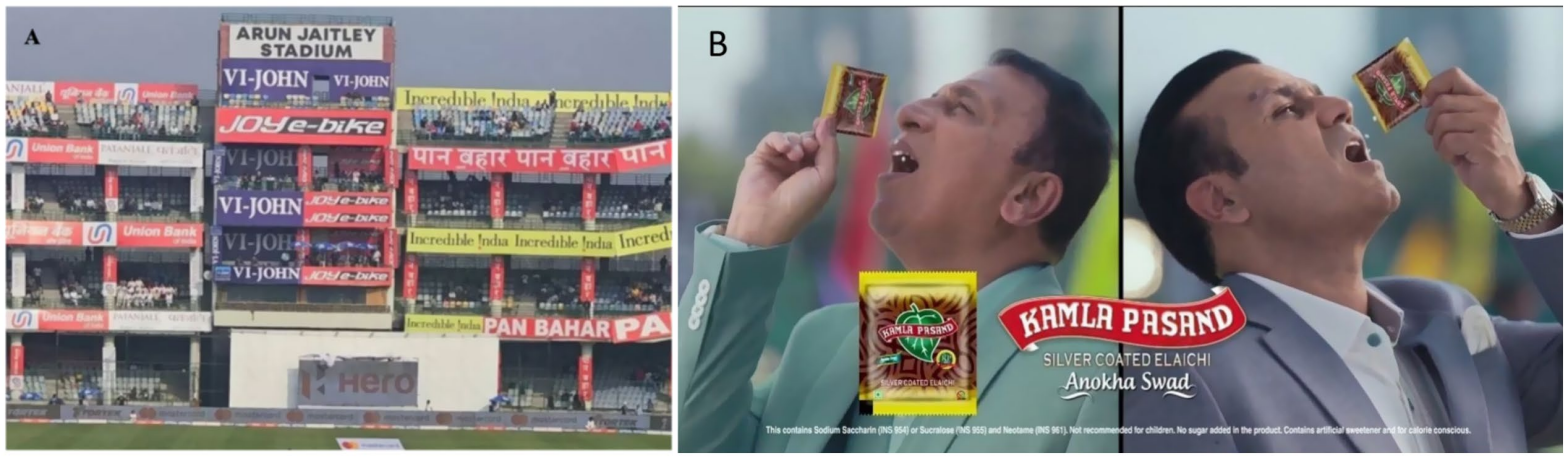
**(A)** Hoardings with smokeless tobacco advertisements in cricket stadium. Reproduced with permission from “Health ministry asks BCCI to ban tobacco ads during cricket matches” by Social Media Dissect. **(B)** Cricketers promoting the use of mouth fresheners which are marketed by pan masala brands. Reproduced with permission from “Sport stars in Kamla Pasand ad: More than a matter of bad taste” by Sanhya Raghavan, Exchange4media.

Bollywood celebrities and cricketers (Figures 4A,B) often endorse these surrogate products, unintentionally contributing to the promotion of harmful substances. While they may not be directly promoting tobacco, their association with these brands lends them credibility and visibility, subtly encouraging tobacco use. This strategy has been criticized for undermining public health campaigns aimed at reducing tobacco consumption and preventing oral cancer. Bollywood celebrities often advocate for healthy lifestyles, yet some, like Shah Rukh Khan, Ajay Devgn, and Akshay Kumar, have faced criticism for endorsing tobacco brands through surrogate advertising. These ads promote products like mouth fresheners to indirectly advertise tobacco, which is legally prohibited. Celebrities such as Amitabh Bachchan and Ranveer Singh have also been associated with pan masala brands, sparking public backlash (25). While some, like Akshay Kumar and Amitabh Bachchan, have since withdrawn from such endorsements, the issue persists in Bollywood and Indian cinema.

Celebrities continue to endorse harmful products, including tobacco-related items through surrogate marketing, primarily due to financial incentives, weak regulations, and cultural normalization. Lucrative endorsement deals from tobacco and surrogate brands make these promotions highly profitable, often outweighing ethical concerns. Many endorsements exploit regulatory loopholes, where tobacco products are indirectly promoted through legal substitutes like pan masala and mouth fresheners. Additionally, marketing agencies and brand strategists prioritize commercial success over credibility, encouraging celebrities to engage in such endorsements despite potential backlash. The social and cultural acceptance of tobacco-related products further reduces the perceived risk of reputation damage, as many fans continue to support their favorite celebrities regardless of ethical contradictions. Unlike in Western countries, organized consumer boycotts and legal consequences remain weak, allowing celebrities to navigate criticism with minimal impact on their careers. Moreover, the influence of the tobacco lobby and industry networks ensures continued celebrity involvement by finding new ways to market these products despite restrictions. As a result, celebrities often endorse both harmful and socially responsible brands simultaneously, relying on short public memory to avoid lasting damage to their image. To curb such endorsements, stronger regulations, stricter enforcement, and public awareness campaigns are necessary to hold celebrities accountable and discourage them from promoting harmful products.

With the growth of social media and digital marketing, India’s youth have become an increasingly accessible target for tobacco industry promotions. A recent report found that 12% of the 2011 documented instances of tobacco marketing over 4 months were surrogate advertisements for smokeless tobacco (29). Data from the Tobacco Enforcement and Reporting Movement (TERM), highlighted that social media platforms like Youtube, Facebook, were used to promote mouth fresheners and pan masala products with branding nearly identical to their tobacco counterparts (30).

Cricket matches, highly popular among the youth, have frequently featured surrogate advertisements for smokeless tobacco products, often during live broadcasts (Figure 4A). These ads, which are promoted under the guise of mouth fresheners or similar products, are frequently endorsed by celebrities. Such promotions during widely viewed sporting events can indirectly appeal to young viewers, making them more likely to associate tobacco use with popular culture and sports, further complicating efforts to reduce tobacco consumption among India’s younger population. A study by the Indian Council of Medical Research (ICMR) and Vital Strategies, published in the British Medical Journal in May, found that 41.3% of all surrogate advertisements for smokeless tobacco brands in 2023 were aired during the final 17 matches of the cricket world cup (31). Another example is that several cricket venues hosting major tournaments like the IPL have featured advertisements for products such as Gutka and Pan Masala. In recent surrogate marketing efforts, public figures promote these brands by endorsing ‘elaichi’ mouth fresheners, which are actually produced by companies that also manufacture tobacco products. These ads are a way to subtly promote tobacco products under the guise of advertising less harmful alternatives, leveraging celebrity influence and sports platforms to indirectly market tobacco-related products.

Efforts to counter surrogate marketing have included stricter regulations and public awareness campaigns, but enforcement remains a challenge. Raising awareness about the harmful effects of such marketing practices and ensuring stricter adherence to advertising bans are crucial in the fight against tobacco use. Given the immense influence these celebrities wield, with millions of fans across the country, the responsibility to make careful choices about the products they endorse lies with them. It is vital that these stars fully understand the impact of their endorsements and consider the consequences of promoting products associated with tobacco. Ideally, they will recognize the weight of their influence and choose to refrain from lending their support to such harmful products, setting a healthier example for their followers.

Tobacco industry’s exploitation of regulatory gaps and ambiguities in tobacco advertising and promotion laws, particularly using social networking sites (SNS) to bypass bans on traditional media advertising (32). Despite progress in tobacco control, including the ban on tobacco advertising under Section 5 of the Cigarettes and COTPA, the industry is circumventing these restrictions through indirect promotion on digital platforms (28). This underscores the urgent need to revisit and strengthen Section 5 of COTPA to address the ambiguities related to SNS and ensure strict enforcement at all levels—national, state, and district. Additionally, empowering the youth by involving them in campaigns aimed at raising awareness and becoming community advocates for tobacco control (30, 33). Engaging young people in such initiatives can help maintain and further the progress made in tobacco control over the past two decades.

### Successful celebrity-driven campaigns in tobacco control and oral cancer prevention

Several public health campaigns in India have successfully engaged celebrities to promote tobacco cessation and oral cancer prevention. These campaigns have been instrumental in raising awareness, especially among young people and rural populations. A study assessing the impact of anti-tobacco advertisements found that a significant proportion of students experienced a positive change in attitude toward tobacco use due to these campaigns. Specifically, 78.35% of male and 90.6% of female students reported a shift in perspective, highlighting the effectiveness of such initiatives in influencing young minds (34). The majority of participants recognized the importance of celebrity involvement in promoting anti-tobacco campaigns. This may be due to the tendency of adolescents to mimic behaviors they observe rather than simply following verbal instructions. Studies have shown that celebrity endorsements of tobacco products in films are causally associated with tobacco use among youth, with a dose–response relationship. This underscores the need for careful consideration of the roles celebrities play in media and advertising (35). The inclusion of a celebrity in the anti-tobacco advertisement enhanced attention and recall among viewers, suggesting that celebrity involvement can be a potent tool in public health campaigns. Post-exposure, 76.1% of participants recalled viewing the anti-tobacco spots during the movie. Recall was notably higher among younger individuals (10–25 years) and rural attendees. However, tobacco users were less likely to recall the messages compared to non-users (72.1% vs. 79.1%) (36). The findings underscore the importance of utilizing mass media, especially with celebrity endorsements, to influence public perceptions and behaviors related to tobacco consumption.

Bollywood legend Amitabh Bachchan has been at the forefront of several public health campaigns in India (37), including those aimed at curbing tobacco use by terminating his contract from pan masala brand (38). His personal decision to quit smoking and his public statements about the harmful effects of tobacco have added authenticity to his involvement in these campaigns. He also expressed his interest in raising awareness about the dangers of both smoking and smokeless tobacco use, particularly targeting rural populations (39, 40). Bachchan’s influence transcends cultural and linguistic boundaries in India, making his participation in such initiatives vital to reaching a broad audience. His involvement in the anti-tobacco campaign will be a prime example of how celebrities can bring attention to critical public health issues and inspire behavioral change at a mass level.

Cricket legend Sachin Tendulkar, one of the most revered sports figures in India, has been involved in multiple public health initiatives, including those aimed at reducing tobacco use. Tendulkar’s endorsement of the anti-tobacco cause is particularly significant in reaching young audiences, especially males, who are at high risk of tobacco addiction and oral cancer. Tendulkar’s involvement helped to counter the glamorization of tobacco use in sports and promoted a healthy, tobacco-free lifestyle (41, 42). His status as a role model for millions of young people has the potential to influence attitudes and behaviors toward tobacco use, especially in communities where cricket holds cultural significance. In southern India, Tamil cinema superstar Rajinikanth has emerged as a powerful advocate for public health, including anti-tobacco initiatives. Rajinikanth’s appeal extends beyond the Tamil-speaking population, and his involvement in health campaigns has resonated with diverse audiences. Rajinikanth’s decision to publicly quit smoking and his active participation in tobacco cessation campaigns has been influential in promoting a tobacco-free lifestyle (43). His endorsement of anti-tobacco initiatives has been particularly effective in reaching middle-aged men, a demographic that often struggles with long-term tobacco addiction.

### The challenges of celebrity involvement in health campaigns

While celebrity involvement in public health campaigns can be highly effective, it is not without challenges. One of the primary concerns is the potential for mixed messaging. Celebrities who endorse tobacco or alcohol products in their past may face credibility issues when they subsequently advocate for health-related causes. For instance, some Bollywood stars who have been associated with tobacco or alcohol advertisements in their earlier careers have faced criticism when they later became involved in anti-tobacco campaigns. Moreover, the authenticity of the celebrity’s involvement is critical to the success of the campaign. Public perception is increasingly shaped by social media, where celebrities are often scrutinized for their personal choices and endorsements. Celebrities who are perceived as insincere or who participate in campaigns solely for financial gain risk undermining the effectiveness of the health message. It is important, therefore, that celebrities who participate in anti-tobacco or oral cancer prevention campaigns demonstrate genuine commitment to the cause, either through personal experiences or long-term advocacy.

Additionally, there is the challenge of ensuring that the message reaches the intended audience. India’s population is diverse, with vast differences in language, culture, and access to media. While Bollywood stars may have national appeal, regional cinema stars and local influencers may be more effective in targeting specific communities, especially in rural areas where tobacco use is most prevalent. For public health campaigns to be successful, they must engage celebrities who resonate with the target demographic, whether through language, cultural affinity, or geographic relevance.

### The role of policy in supporting celebrity-led health campaigns

While celebrity-driven campaigns can raise awareness and encourage behavioral change, they must be supported by robust public health policies to ensure long-term impact. In India, anti-tobacco legislation, including the Cigarettes and Other Tobacco Products Act (COTPA), has laid the foundation for tobacco control. However, enforcement remains inconsistent, particularly in rural areas where smokeless tobacco is more prevalent. Celebrity endorsements of anti-tobacco campaigns can help to reinforce the importance of these policies by making the public aware of existing regulations, such as bans on tobacco advertising, pictorial health warnings on tobacco products, and smoke-free zones. However, for these campaigns to be truly effective, they must be coupled with stronger enforcement of tobacco control policies, increased access to smoking cessation resources, and greater investment in public health infrastructure.

In addition to policy enforcement, education plays a crucial role in tobacco prevention. Schools and community programs that promote tobacco-free lifestyles, combined with celebrity-led campaigns, can create a multi-faceted approach to tobacco control. For instance, celebrities can participate in educational programs that target young people particularly in high-risk areas, to foster early prevention and reduce the likelihood of tobacco addiction later in life.

### Cessation strategies for smokeless tobacco users in India

Smokeless tobacco (SLT) use in India is driven largely by nicotine addiction, the primary active substance in tobacco. Though nicotine absorption is slower with SLT than smoking, its peak levels are similar, leading to sustained addiction. The criteria for nicotine dependence are based on continued use despite knowing its risks. Cessation efforts include Tobacco Cessation Clinics (TCCs), established in 2002, where behavioral counseling and pharmacotherapy are offered. Despite these efforts, the quit rate remains at around 31% (44). A cross-sectional household survey conducted in Jamul, Chhattisgarh, among 450 individuals aged 35–44 and 65–74 years, aimed to assess the prevalence and quit behavior associated with smokeless tobacco (SLT) use. Among the participants, 61.1% were male and 38.9% female. The prevalence of SLT use was high at 67.8%, with 59.1% of users reporting daily consumption. Gutkha was the preferred form among middle-aged individuals, whereas khaini was more common among the older adult. Other frequently used products included paan with tobacco and gudakhu, the latter cited by 71.34% of users for oral hygiene and digestive ease. Television broadcasts and warning labels on SLT packages emerged as effective tools for awareness. However, many users attempting to quit without support experienced withdrawal symptoms, posing major barriers to cessation. The study emphasizes the need for regionally tailored awareness campaigns through television, professional counseling, pharmacotherapy, and peer support to reduce SLT use and improve quit rates in the community (45). A secondary analysis of Global Adult Tobacco Survey data from 2009–2010 (GATS-1) and 2016–2017 (GATS-2) examined trends in smokeless tobacco (SLT) cessation behavior in India. While SLT use declined from 25.9 to 21.4%, the proportion of users attempting to quit in the past year also slightly dropped from 33.7 to 32.0%. Similarly, the intention to quit within the next 12 months decreased markedly from 27.2 to 19.4%. Daily smokers using SLT had significantly lower odds of quitting, whereas higher education was positively associated with cessation efforts. Despite the reduction in use, the uptake of cessation aids like counseling, nicotine replacement therapy, and mCessation remains limited, underscoring the need to strengthen awareness and cessation infrastructure in India (46). The willingness to quit tobacco in the near future, along with past quit attempts, are critical aspects of cessation behavior that contribute significantly to achieving successful tobacco cessation. Developing targeted tobacco control policies tailored to specific user groups can help close the gap between tobacco users and available cessation services. Given the wide variation in quit attempt rates across regions, there is a pressing need for the rigorous enforcement of tobacco control measures and intervention programs nationwide. A recent study, using GATS-2 (2016–17) data, analyzed factors influencing quit attempts among Indian tobacco users through complex sample logistic regression. The quit attempt rate was 33.7% for smokers and 28.9% for smokeless tobacco (SLT) users, with significant variation across states (*p* < 0.001). Higher odds of quit attempts were seen among individuals from scheduled castes and other backward classes. For SLT users, age, social group, and awareness of health risks were significant predictors. Notably, most users—83.5% of smokers and 88% of SLT users—attempted to quit without any formal cessation support. These findings highlight the need for targeted, group-specific tobacco control policies and improved access to cessation services across India (47). Therefore, additional interventions include mass media, workplace campaigns, and community programs could aid SLT cessation.

### Advocacy and policy measures to reduce smokeless tobacco use

India has implemented several policies to curb smokeless tobacco use, notably through COTPA of 2003, and specific state and court orders, which have been pivotal in banning products like gutka. Government leadership, coupled with NGO advocacy, has driven these reforms. Initiatives like public interest litigation (PIL), media advocacy, and raising awareness about SLT’s health risks have strengthened enforcement efforts. However, challenges remain in areas such as disposing of seized products, preventing smuggling, and stopping surrogate advertising.

The Ministry of Health and Family Welfare (MoHFW) has also advised states to raise taxes on tobacco products, while multi-stakeholder consultations work to improve law enforcement (48). Despite bans on gutka in most states, issues like interstate smuggling and the sale of split packets still persist. Public health consultations and initiatives, such as the first National Consultation on Smokeless Tobacco, emphasize the need for continued efforts in tobacco control. Effective enforcement, improved cessation services, and combating industry interference are essential for more comprehensive control of SLT products.

The tobacco industry frequently contests tobacco control policies in court, but the Indian government, supported by civil society, has successfully defended many regulations. Court rulings have been pivotal in prohibiting tobacco in toothpastes (1992), banning gutka and pan masala in plastic sachets (2011), and restricting tobacco advertisements and sponsorships (2012–2013). The Food Safety and Standards Act of 2006 further banned tobacco as a food ingredient, leading to widespread gutka bans in 2012. The judiciary has continued to monitor compliance, seeking reports on enforcement from states.

**Figure 5:**
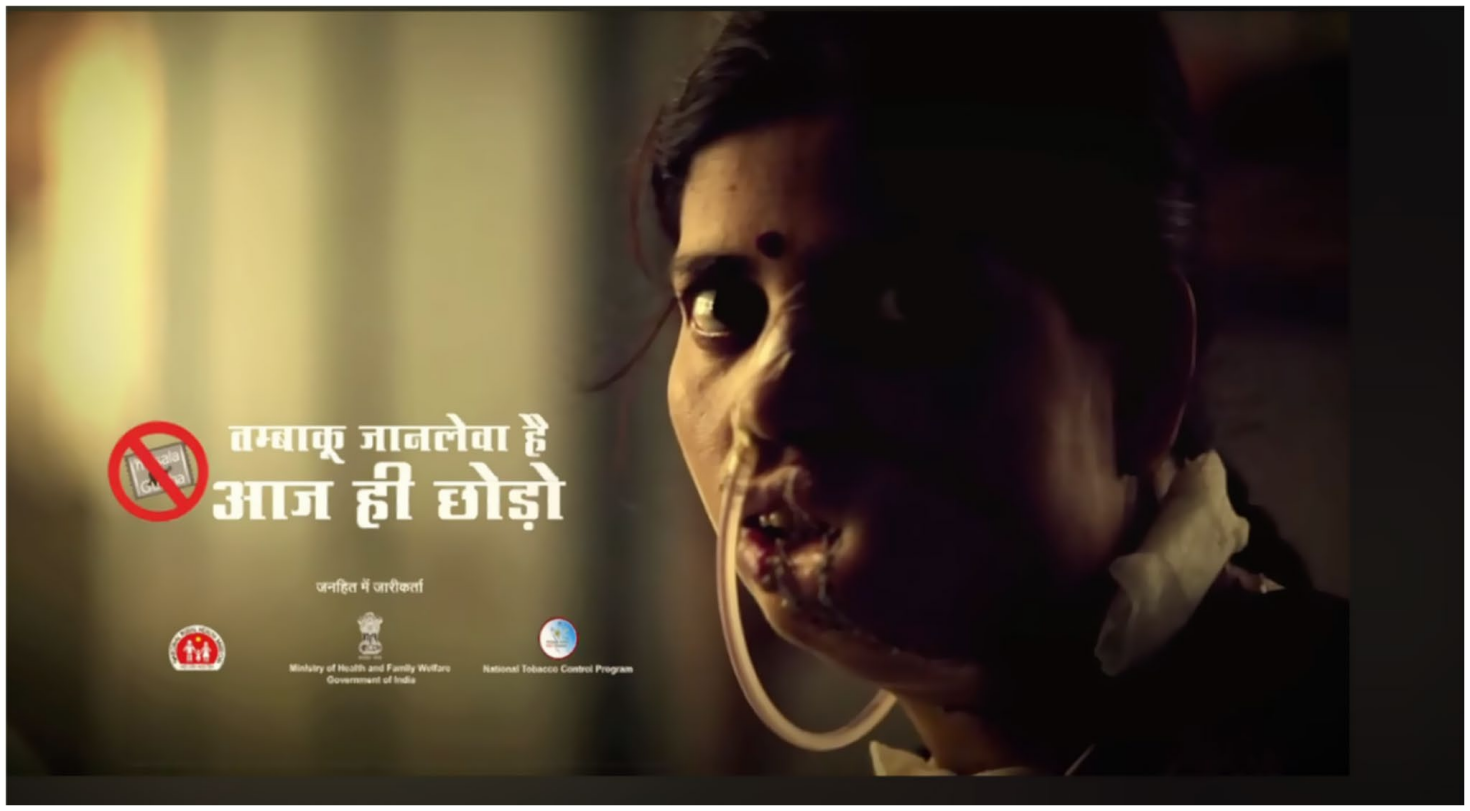
Image from the personal testimony of Ms. Sunita Tomar. The advertisement shows Sunita before and after an operation to remove the cancerous growth and a part of her mouth. This Public Service Announcement (PSA) has been developed by the Ministry of Health and Family Welfare, India with technical assistance from World Lung Foundation. Screenshot from: “India - Sunita (English) - Testimonial” by Vital Strategies.

In a new initiative aimed at curbing tobacco use, the Indian government is seeking to make anti-tobacco advertisements mandatory on over-the-top (OTT) platforms (49). Under this proposal, viewers would be required to watch a 30-s anti-tobacco ad as soon as they open an OTT app, and crucially, the ad would be unskippable. This move is part of a broader effort by the government to reduce tobacco consumption and raise awareness about its harmful effects, particularly given the increasing popularity of OTT platforms among younger audiences (49). These platforms, such as Netflix, Amazon Prime, and Disney+ Hotstar, have become major channels for content consumption in India, making them an ideal medium for delivering health-focused messages. By ensuring that all users are exposed to anti-tobacco messages, this policy aims to promote public health and deter tobacco usage across all age groups. The Government is dedicated to providing healthcare and social security to all citizens of India. A crucial aspect of its strategy is raising awareness about the harmful effects of tobacco through mass media campaigns, the Film Rule, and various other Behavior Change Communication methods. New videos promoting awareness about oral cancer and the importance to quit smokeless tobacco products is promoted (Figure 5).

### Comparative analysis of smokeless tobacco cessation strategies: India vs. the United States

Smokeless tobacco (SLT) use poses significant public health challenges in both India and the United States, albeit with differing prevalence rates, cultural contexts, and cessation strategies. A comparative assessment of SLT cessation efforts in these two countries offers valuable insights into policy effectiveness, implementation gaps, and opportunities for cross-learning. In India, approximately 21.4% of adults use SLT products, with higher prevalence among men, rural populations, and those with lower educational attainment, as reported in the Global Adult Tobacco Survey 2016–17 (50). In contrast, the United States has a lower SLT prevalence of around 2.4%, with use more common among men, young adults aged 18–25, and rural populations (51).

India’s primary legislative tool, the Cigarettes and Other Tobacco Products Act (COTPA), 2003, mandates pictorial warnings, advertising bans, and restrictions on tobacco sales near educational institutions. Additionally, several states have imposed bans on gutkha and pan masala under food safety laws; however, enforcement remains variable (52). The United States enforces tobacco regulation through the Family Smoking Prevention and Tobacco Control Act, granting the FDA authority over SLT products, including warning labels, advertising restrictions, and pre-market approvals for modified-risk products (51).

India offers cessation support through Tobacco Cessation Clinics (TCCs) and the mCessation program, which delivers mobile-based quit support. Despite these measures, service uptake is low—GATS-2 data show that over 88% of SLT users attempt to quit without professional help (1). A feasibility trial of text message–based cessation intervention in India further highlights the potential but underutilized nature of digital support (52, 53). In comparison, the U.S. provides structured cessation pathways, including a national quitline (1-800-QUIT-NOW), behavioral counseling, and access to FDA-approved pharmacotherapies such as nicotine replacement therapy (NRT), bupropion, and varenicline (54). Recent interventions also include text-based cessation support for rural users (55).

Public awareness campaigns in the U.S., like the CDC’s “Tips From Former Smokers,” have played a pivotal role in increasing risk perception and quit intentions among SLT users (53). Conversely, in India, SLT use is culturally ingrained and often perceived as traditional or medicinal (e.g., gudakhu for oral hygiene). Though pictorial warnings and TV-based campaigns exist, they have yet to create substantial behavioral change, particularly among long-term users (56).

While both countries have taken steps to curb SLT use, the U.S. exhibits a more integrated and better-funded cessation framework, complemented by strong public education and centralized enforcement. India’s initiatives—such as COTPA and mCessation—are commendable, but often fall short due to weak implementation, fragmented services, and cultural normalization of SLT. However, India’s mCessation model offers a scalable, low-cost solution that could be adapted for underserved U.S. populations. Adopting proven strategies from the United States such as consistent policy implementation and emotionally driven anti-tobacco messaging could strengthen India’s approach to reducing SLT-related diseases.

### Comprehensive strategies for celebrity-driven tobacco control and oral cancer prevention in India

The role of celebrities in promoting oral cancer prevention and limiting tobacco use in India is clear, but to achieve lasting change, a more comprehensive approach is required. This includes sustained engagement from celebrities, policy support, community involvement, and the integration of technology and social media to reach a broader audience.

#### Sustained celebrity engagement

While one-off endorsements from celebrities can create temporary spikes in awareness, long-term engagement is necessary to sustain public interest and drive behavioral change. Celebrities who are consistently involved in public health campaigns, and who demonstrate personal commitment to the cause, are more likely to have a lasting impact. For example, continuous participation in anti-tobacco initiatives, coupled with regular public appearances, social media engagement, and partnerships with health organizations, can reinforce the message and create a broader cultural shift away from tobacco use.

#### Community-based campaigns

Celebrity involvement should also be complemented by grassroots efforts that engage local communities in the fight against tobacco use. Community health workers, local leaders, and regional celebrities can work together to create culturally relevant campaigns that resonate with specific populations. This approach is particularly important in rural areas, where tobacco use is deeply embedded in cultural practices and where access to mainstream media may be limited.

#### Leveraging social media and technology

Advertisements wield significant influence, and when celebrities are involved, their impact intensifies. Pro-tobacco ads featuring well-known figures can encourage tobacco use by glamorizing it, particularly among younger audiences. However, the same power can be harnessed for anti-tobacco campaigns. When celebrities endorse anti-tobacco messages, they not only raise awareness but also set an example, motivating fans to quit or avoid starting tobacco use. Their public image and large following can amplify the message, making it more relatable and convincing, ultimately driving more significant social change against tobacco consumption. In the digital age, social media platforms provide an unprecedented opportunity to reach millions of people with targeted health messages. Celebrities who actively engage with their followers on social media can use these platforms to promote tobacco cessation and oral cancer prevention. Viral campaigns, challenges, and interactive content can further enhance the reach and impact of these messages, particularly among younger audiences.

### Policy recommendations- limiting celebrity involvement to prevent smokeless tobacco use in India

Policy recommendations regarding celebrity involvement in surrogate marketing of smokeless tobacco products should emphasize stricter regulation and enforcement (Table 2). This includes banning celebrity endorsements of surrogate products like pan masala when linked to tobacco, imposing penalties for violations, and mandating clear disclosures about the association between such products and tobacco. Engaging celebrities in public health campaigns, especially anti-tobacco initiatives, can help shift public perception. Additionally, targeted restrictions on youth platforms and monitoring systems should be put in place to prevent harmful messaging and increase accountability. We have provided out inputs to frame policies.

**Table 2:** Policy recommendations to regulate surrogate marketing of tobacco products.

Stricter regulations on endorsements
Implement stringent regulations that prevent celebrities from endorsing surrogate products like mouth fresheners and pan masala when these brands are tied to tobacco products. A clear definition of what constitutes surrogate marketing must be established and enforced.
Penalties for violations
Introduce legal and financial penalties for celebrities and companies involved in promoting smokeless tobacco through surrogate advertising, deterring them from participating in such activities.
Awareness programs for celebrities
Launch educational initiatives aimed at celebrities to raise awareness about the public health impact of their endorsements, emphasizing their responsibility in influencing public behavior and preventing tobacco use.
Mandated disclaimers in ads
Require any advertisement for products associated with tobacco companies to carry clear disclaimers about their connection to tobacco products, even if surrogate products are being advertised.
Engage celebrities in anti-tobacco campaigns
Encourage celebrities to take part in anti-tobacco initiatives, using their influence to counter the promotion of harmful products and shift public perception away from tobacco use.
Collaboration with NGOs
Lawmakers and stakeholders should collaborate with non-governmental organizations to create campaigns that highlight the manipulative nature of surrogate advertising, aiming to reduce public acceptance of smokeless tobacco products.
Monitoring and enforcement
Establish a monitoring system to track surrogate advertising, particularly during high-visibility events such as sports matches, and ensure swift action against any violations of advertising laws.
Public recognition for responsible celebrities
Establish programs to publicly recognize and reward celebrities who choose to promote health and wellness products, encouraging a shift in the endorsement landscape.

## Conclusion

Community media, particularly audio-visual platforms, offer broader reach and greater cost-effectiveness compared to interpersonal communication. Mass media campaigns with health-focused messages have effectively influenced diverse populations, including shaping social norms and attitudes toward smokeless tobacco use. These campaigns also serve as powerful advocacy tools for public policy development. A comprehensive strategy that integrates various media platforms can amplify the impact by reinforcing consistent health messages across multiple channels, thereby enhancing public awareness and supporting tobacco control efforts. Celebrities have the potential to play a transformative role in the prevention of oral cancer and the reduction of tobacco use in India. Their influence, particularly in a media-driven society, can amplify public health messages, challenge social norms around tobacco use, and encourage behavioral change. However, for these campaigns to be truly effective, they must be carefully crafted, culturally sensitive, and supported by robust public health policies and community engagement. By leveraging the power of celebrity alongside comprehensive tobacco control policies and public health education, India can make significant strides in reducing the burden of oral cancer and improving the health and well-being of its population.
